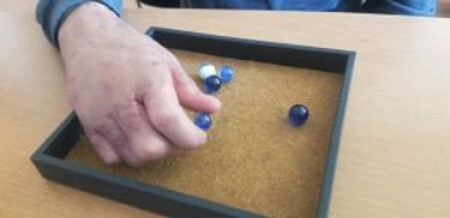# 32 HANDFULS: A New Clinically Feasible Measure of Hand Outcome for Children with Burns and Trauma

**DOI:** 10.1093/jbcr/irae036.032

**Published:** 2024-04-17

**Authors:** Ingrid Parry, Michelle James, Machelle Wilson, David G Greenhalgh

**Affiliations:** Shriners Hospitals for Children, Carmichael, California; Shriners Hospital for Children, Northern California, Sacramento, California; University of California Davis, Sacramento, California; Shriners Hospitals for Children, Carmichael, California; Shriners Hospital for Children, Northern California, Sacramento, California; University of California Davis, Sacramento, California; Shriners Hospitals for Children, Carmichael, California; Shriners Hospital for Children, Northern California, Sacramento, California; University of California Davis, Sacramento, California; Shriners Hospitals for Children, Carmichael, California; Shriners Hospital for Children, Northern California, Sacramento, California; University of California Davis, Sacramento, California

## Abstract

**Introduction:**

Fine motor dexterity is important for children's normal hand function and development. In-hand manipulation (the collection and repositioning of items in the hand) and palmar workspace (functional volume in the palm) are two aspects of hand function that often become simultaneously impaired after hand trauma or burn injury. Rehabilitation emphasizes recovery of these hand functions yet few functional outcome measures address both of these constructs and even fewer are appropriate for children.

**Methods:**

This study developed a new hand outcome measure to specifically assess these constructs and tested it on 192 children between the ages of 2-21. The test, called ‘HANDFULs’, uses simple instructions and inexpensive materials (i.e. marbles) shown in Figure 1. Means, standard deviations, and 95% confidence limits were estimated for children in seven age groups. Associations between age and the HANDFULs variables were examined using one-factor ANOVA. Multiple regressions were fit to test for associations between age and the HANDFULs variables after controlling for height, hand size, and hand strength. A stepwise selection procedure was then implemented, holding age into the model, to remove otherwise non-significant variables. Test-retest reliability was tested with the Shrout Fleiss Reliability Index and significance was set at p< 0.05.

**Results:**

Expected normal values are reported in Figure 2 for palmar workspace volume (marbles that fit in the hand) and in-hand manipulation (collection time per marble) by age group. Age (0.16 (0.06), p=0.009), hand span (0.47 (0.09), p< 0.0001) and hand width (0.9 (0.29), p=0.003) were significantly associated with number of marbles that fit in the hand, representing palmar workspace capacity. Although hand strength and size characteristics were tested, only age (-0.02 (0.01), p=0.001) was significantly associated with the HANDFULs score of time per marble. The Intraclass Correlation Coefficient was 0.94 demonstrating excellent test-retest reliability.

**Conclusions:**

This study developed a new pediatric hand outcome measure to address hand function to address a gap related to burn and trauma recovery. The measure was tested on able-bodied, noninjured children to establish validity and reliability, provide reference values for patients with injuries by age groups, and is clinically feasible to administer.

**Applicability of Research to Practice:**

Outcome test for burn injured hands.